# Case report and review of the literature: a unique presentation of Blau syndrome in a Palestinian family

**DOI:** 10.3389/fped.2025.1482846

**Published:** 2025-07-22

**Authors:** Sandra Subhi Hnaihen, Nofouz I. A. Maswada, Aya Ahmad Bahar, Mayar Idekedek, Fawzy M. Abunejma

**Affiliations:** ^1^ Medical Research Club, Faculty of Medicine, Al-Quds University, Jerusalem, Palestine; ^2^ Head of Pediatrics Department, Faculty of Medicine, Hebron University, Hebron, Palestine

**Keywords:** Blau syndrome, renal failure, *NOD2* gene, polyarthritis, Palestine

## Abstract

Blau syndrome (BS) is a rare inherited systemic disorder, attributed to a gain-of-function mutation in the nucleotide-binding oligomerization domain (*NOD2*) gene, which results in the upregulation of pro-inflammatory cytokines. This syndrome was initially described as a classic triad of arthritis, dermatitis, and uveitis. In this article, we report a unique presentation of renal failure in a 13-year-old patient who was diagnosed with BS. Interestingly, the patient had only displayed one of the three classical signs, i.e., arthritis. In our case, she had never had any symptoms of the skin or ocular involvement and had just developed arthritis. As a result, the patient was initially misdiagnosed as a case of Juvenile Idiopathic arthritis (JIA). Hence, it's crucial to consider other possible diagnoses when JIA cannot fully explain the patient's presentation and whenever there's an atypical response to treatment. Furthermore, a detailed family history and further investigations; such as genetic testing may be essential for the diagnosis of BS.

## 1 Introduction

Blau syndrome (BS) was first described in 1985 and is a rare, hereditary auto-inflammatory disorder. It is characterized by a triad of granulomatous polyarthritis, dermatitis, and uveitis. This condition is estimated to affect 1 in 1 million children (1). Throughout the years, there have been almost 200 reported cases of BS worldwide (2). The syndrome's clinical presentation was attributed to the gain-of-function mutation in the nucleotide-binding oligomerization domain (*NOD2*) gene found on chromosome 16 (3, 4). There have been at least 30 known mutations in the *NOD2* gene, most commonly near the NOD/NACHT domain (5–13). This gene plays a critical role in the activation of the nuclear factor-κB pathway. The presence of mutations in the *NOD2* gene results in the overstimulation of these signaling pathways which ultimately results in the overproduction of inflammatory markers leading to increased inflammation and granuloma formation (14).

*NOD2*-associated disease, referred to as BS in familial cases and early-onset sarcoidosis in sporadic cases, results from gain-of-function mutations in the *NOD2* gene on chromosome 16. It is characterized by a triad of granulomatous arthritis, uveitis, and dermatitis. Due to overlapping clinical features with juvenile Idiopathic arthritis (JIA), particularly in the early stages, differentiation can be challenging and may lead to diagnostic delays or misclassification (15–20).

Steroids are considered as the main treatment for BS. As for patients not responding to steroids, biological drugs, such as anti-TNF, methotrexate (MTX), and interleukin-1 inhibitors, are described as alternatives in previous lectures (21–23).

Our patient was initially diagnosed with JIA, but later on, she developed renal failure, along with a detailed review of the family history which revealed similar clinical manifestations in both the father and a brother—raising a strong suspicion of an underlying hereditary condition. Genetic testing of both her and her affected family members confirmed her diagnosis of BS. The rarity of this disease and the atypical presentation of our patient made it worthwhile to publish.

## 2 Case presentation

A 13-year-old girl was presented to our outpatient clinic at the age of 11 years old with painful joint swelling for 6 years. Her long-term history of arthritis could be traced back to when she started to suffer from symmetrical polyarthritis at the age of 5 years old, which was in both ankles and wrists and was associated with morning stiffness. Afterward, she was presented to a rheumatologist and was diagnosed with JIA. Hence, she was treated with oral prednisone (6 mg) and MTX, with a significant improvement in the joints’ swelling and pain with oral prednisone (6 mg), although the patient had no complaints. Unfortunately, during the following 6 years, her symptoms were not well-controlled.

In January 2021, at 11 years old, she presented to our hospital for the first time due to recurrent arthritis in the absence of fever, skin rash, or any other systemic manifestations. Physical examination revealed flexion contracture of proximal interphalangeal (PIP) and metacapo-phalangeal (MCP) joints of thumb and index fingers at both hands, hyperextensibility at distal interphalangeal joints (DIP) bilaterally, boggy arthritis at both wrists and ankles, and cystic swelling on the dorsal surface of the hands as mentioned in Table 1. On the other hand, an ophthalmic examination revealed no evidence of uveitis.

**Table 1:** Timeline event.

Date	Event
–/2015	Misdiagnosed as JIA, treated with oral prednisone(6 mg) and MTX
January 2021	Hand and feet radiographs: periarticular osteopenia mainly at MCP and PIP joints with erosions and deformity
January 2021	Ophthalmic examination: no evidence of uveitis
May 2021	Intra-articular injection of triamcinolone acetonide 20 mg in each wrist and ankle joints without immediate complications
September 2023	Abdominal ultrasound: revealed a normal-sized kidney with increased echogenicity and an enlarged spleen (13 cm)
September 2023	Exome sequencing analysis: pathogenic variant in the *NOD2* gene (NM_001370466.1 16:50744823 c.920G>A p.Arg307Gln) and confirmed the diagnosis of BS
September 2023	Subcutaneous injection of adalimumab (40 mg every 2 weeks), subcutaneous injection of abitrexate (15 mg weekly), and prednisone of low to moderate dose (9 mg); if relapsed, the dose was increased to 15 mg daily for 10 days
December 2023	The patient had shown significant improvement, along with decreasing creatinine levels

It is worth mentioning that the patient's father has been a known case of rheumatoid arthritis for 30 years, with contractures at PIPs on both hands and hyperextensibility at DIPs bilaterally. He reported that he had the same swelling at both wrists and ankles during his childhood; however, he displayed no skin or ocular symptoms. Her brother, aged 9 years old, had developed the same presentation and joint changes in the absence of skin and ocular symptoms.

The patient's laboratory results were hemoglobin 11.3 g/dl, mean corpuscular volume (MCV) 60, white blood cell count (WBC) 7,400/mm^2^, and platelet count 334,000/mm^2^, while kidney and liver function tests, lipid profile, erythrocyte sedimentation rate (ESR) and C-reactive protein (CRP) were within normal ranges. Testing for hepatitis B surface antigen (HBsAG) and anti-hepatitis C virus antibodies were non-reactive. Her rheumatoid factor (RF), anti-nuclear antibody (ANA), HIV, and PPD tests were negative. Hands and feet radiographs showed periarticular osteopenia mainly at MCP and PIP joints with erosions or deformity. She was started on a TNF-α inhibitor (Enbrel) through a subcutaneous injection (35 mg every week) for 4 months. At that time, she showed partial improvement, but the swelling persisted. As a result, she was given an intra-articular injection of triamcinolone acetonide 20 mg in each wrist and ankle joints with no immediate complications, and she was continued on Enbrel (25 mg weekly).

During the following 2 years, she showed a poor response to Enbrel; on the other hand, she showed a good response to a low-dose prednisone regarding pain and swelling.

**Figure 1:**
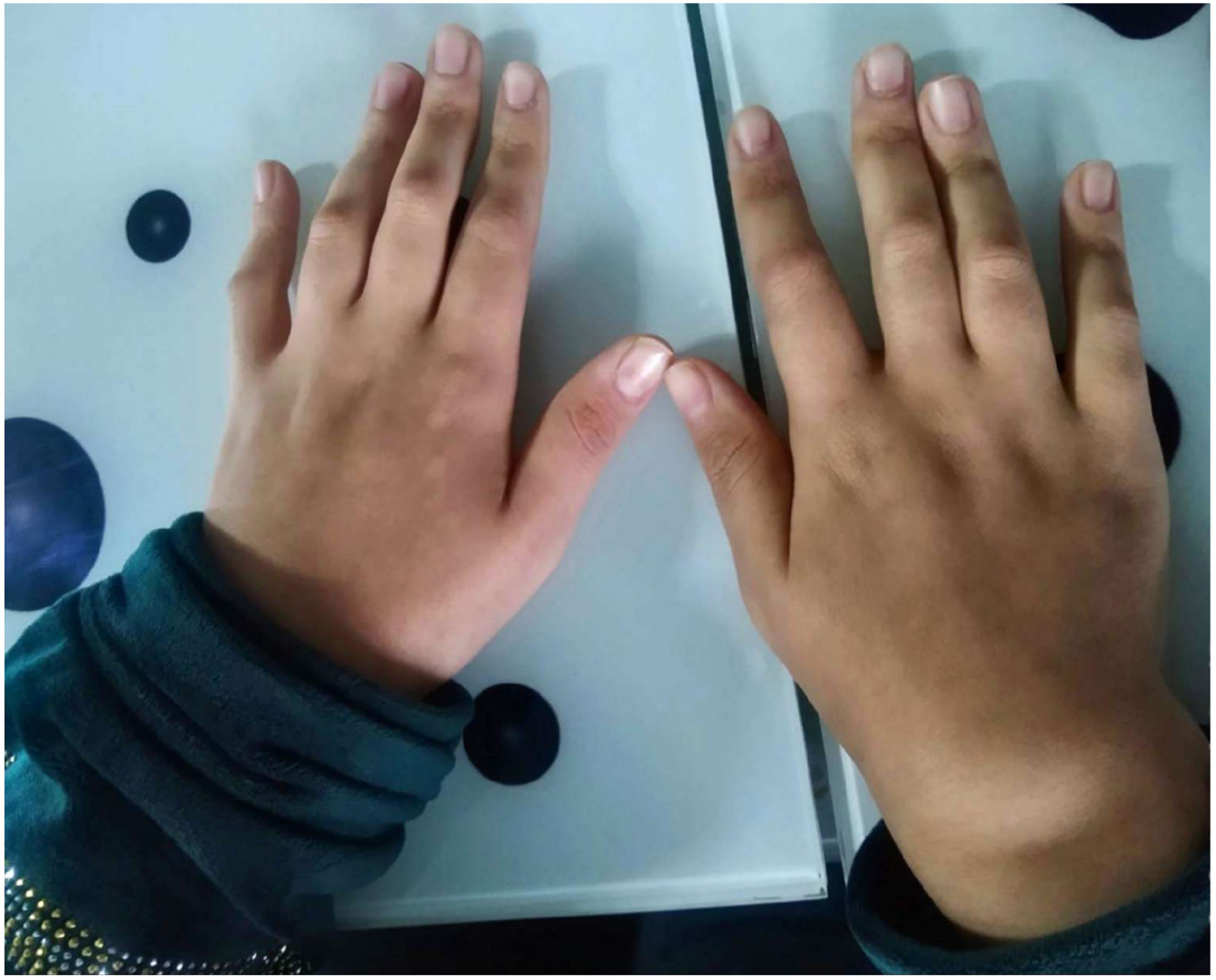
Hands deformity showing camptodactyly of all fingers at both hands.

In a follow-up visit during September 2023, she still had boggy arthritis in both wrists and ankles joints, associated with tender limitation of movement of elbows and progressive camptodactyly of all of her fingers at both hands (Figure 1). No swelling or tenderness in other joints (hips, shoulders, and small joints of feet) and skin rash were noticed.

Laboratory tests were obtained and showed hemoglobin 7.8 g/dl, MCV 65, WBC count 8,200/mm^2^, and platelet count 377,000/mm^2^, and kidney function test showed elevated creatinine (1.9) and elevated BUN (25), while lipid profile, liver function test, liver enzymes, thyroid function test, iron study, electrolytes, fasting blood sugar, hemoglobin A1C, uric acid and antistreptolysin O test were all within normal ranges.

Based on the abovementioned laboratory results, her microcytic anemia was initially diagnosed as anemia of chronic disease. Moreover, an abdominal ultrasound was done and revealed a normal-sized kidney with increased echogenicity and an enlarged spleen (13 cm).

Considering the aforementioned clinical manifestations, including recurrent boggy arthritis with camptodactyly and renal insufficiency, and due to poor response to three lines of disease-modifying antirheumatic drug (DMARDs) and similar family history of the same presentation, an exome sequencing analysis for the patient, her father, and her brother was performed. It revealed the presence of a pathogenic variant in the *NOD2* gene (NM_001370466.1 16:50744823 c.920G>A p.Arg307Gln), according to ClinVar. This result confirmed the diagnosis of BS in all family members. It is worth mentioning that they had another mutation in the HBA1 (c.326delC); this indicated that she, her father, and her brother are also carriers for alpha thalassemia, which explained the patient's microcytic anemia. However, it has not been described in the literature that these mutations result in direct dermatological, ocular, or renal manifestations.

After the patient was successfully diagnosed with BS, her treatment was switched from Enbrel (25 mg weekly) to subcutaneous injection of adalimumab (40 mg every 2 weeks), subcutaneous injection of abitrexate (15 mg weekly), folic acid (5 mg weekly) 1 day after abitrexate, and prednisone of low to moderate dose (9 mg). She was recommended to increase the dose to 15 mg daily for 10 days if a relapse occurs.

The patient subsequently showed significant improvement, along with decreasing creatinine levels. A regular ophthalmic examination was recommended to rule out uveitis, which was free.

## 3 Discussion

BS is a rare, dominantly inherited auto-inflammatory granulomatous disorder that is caused by gain-of-function mutations in the *NOD2* gene (11). The *NOD2* gene is located on chromosome region 16q12.1–13 (6), which includes leucine-rich repeats (LRRs), an N-terminal caspase recruitment domain. This gene is necessary for identifying muramyl dipeptide (MDP), which in turn activates the nuclear factor-κB (NF-κB) pathway (11, 23–25). The synthesis of pro-inflammatory molecules, such as IL-1b, IL-6, IL-18, and TNF-α, is increased via this pathway, causing autoimmune inflammatory reactions and non-caseous granuloma formation (11).

Hence, it is essential to understand the pathophysiology of BS to establish effective management strategies for this disorder. Many mutations in the *NOD2* gene were described, with R334Q and R334W mutation responsible for the most reported cases, occurring in 40%–80% of patients (8–10, 12, 26). Our patient's genetic testing showed the R334Q/R307Q mutation, which is a known *NOD2* gene missense mutation that has been found in patients with BS. To avoid potential confusion, it is important to state that the c.920G>A (p.R307Q) variant described using NM_001370466.1 corresponds to the c.1001G>A (p.R334Q) variant when using the commonly referenced NM_022162.3 transcript, which has been widely used in the literature on Blau syndrome.

The typical triad of BS includes recurrent non-caseating granulomatous dermatitis, symmetrical arthritis, and uveitis, which usually presents before the age of 3–5 years (27). Among these classical triads, the earliest presentation is usually related to skin manifestations, including erythema with a maculopapular small scaly pattern on the trunk and extremities and a papulo-nodular reddish rash accompanied by several firm subcutaneous nodules (23, 27).

Moreover, joint manifestation is the second earliest presentation of BS, including symmetrical polyarthritis, and usually presents before 10 years old. The usually affected joints are the wrists, interphalangeal joints, MCP joints, elbow, ankles, and knees (10), in addition to camptodactyly which can be usually seen in approximately 60% of BS patients. The joints are described as “boggy” due to the presence of teno-synovial cysts (8, 11, 12).

As for ocular manifestations, they present in approximately 80% of patients, with a median age of onset of 4.4 years. Those are usually bilateral uveitis starting as posterior or anterior uveitis, leading to panuveitis. Bilateral panuveitis is the most common presentation (8, 10, 28).

As a result, in the absence of this triad, recognition of BS may be challenging. According to recent research, there was a significant prevalence of sporadic instances, and the majority of patients had fewer than three of the diagnostic criteria. In this paper, we report a 13-year-old patient female with early-onset systemic polyarthritis, at 5 years old, who presented with swollen joints of her hands and feet without the classic manifestation of BS or any other systemic manifestations. Physical examination showed swollen deformed wrists with cystic swelling on the dorsum surface of hands and ankles. Accordingly, she was misdiagnosed with JIA. An ophthalmic exam to look for uveitis was conducted every 4 months with no detected abnormalities, and without any history of skin lesions decmonted. At the age of 13 years old, the progression of arthritis resulted in contractures at PIP, hyperextensibility at DIP, and Bouchard's arthropathy of small joints of hands with tender limited motion at elbows.

In addition to the classic triad, BS patients may present with atypical manifestations related to other organ systems, including cardiovascular, renal, and central nervous systems (11, 23). The extent of the granulomatous inflammation may involve single organs, such as the liver, spleen, kidney, lymph node, or parts of vessels (23). Vascular involvement is a rare manifestation of BS, including large and medium vessels. Histologic studies of biopsied lesions showed non-caseating granulomas with multinucleated giant cells. To the best of our knowledge, over the last 15 years, only 10 patients with BS have been documented in the English literature with renal involvement. All reviewed cases are summarized in Table 2. In our case, at 13 years old, the patient developed renal failure, with a creatinine value of 1.9 mg/dl. The emergence of this new symptom directed us toward other systemic diseases. A thorough medical history, focused on other family members, revealed a similar presentation of polyarthritis in her father and brother, which guided us toward genetic counseling and testing.

**Table 2:** Summary of reported Blau syndrome patients with renal involvement.

Reference	Year	Age	Sex	Age of onset	Rash	The first presentation	Eye manifestation	Arthritis	Fever	Renal involvement	Affected parents
(30)	2016	17 months	Male	11 months	Yes	–	No	No	Yes	Yes	No family history
(31)	2021	12 years	Female	3 years	No	–	Yes	Yes	No	Yes	No family history
(32)	2021	23 years	Female	3 years	Yes	Fever and arthritis	Yes	Yes	Yes	Yes	No family history
(33)	2023	20 years	Female	2 years	Yes	Rash once at 10 months of age, arthritis, and recurrent fever	Yes	Yes	Yes	Yes	No family history
(34)	2019	41 years	Female	8 years	Yes	Rash and severe headache	Yes	Yes	No	Yes	Positive family history in the patient's sister and brother
(14)	2019	10 years	Female	2 years	No	Arthritis	No	Yes	No	Yes	No family history
(35)	2016	4 years	Female	7 months	Yes	-	Yes	Yes	Yes	Yes	Positive family history in the patient's sister and father
(36)	2010	2.5 years	Female	5 months	Yes	Rash all over her body	No	Yes	Yes	Yes	Positive family history
(37)	2019	48 years	Female	–	Yes	Arthritis	No	Yes	No	Yes	Affected two children
(38)	2017	45 years 24 years 18 years	Female Female Male	2 years 3 years 1 year	– Yes Yes	– Arthritis and rash Arthritis and rash	– No No	Yes Yes Yes	– – –	Yes No No	First patient is has affected two children

Another educational value that makes our case unique is her presentation of renal failure in the absence of the classical clinical triad of BS. Our patient initially presented with arthritis, later on, she developed renal failure within a span of 4 years, and up to this date of writing this manuscript, she has not developed any skin or ocular manifestations.

While it is crucial to control joint and ocular involvements for improving the prognosis of BS, high-dose corticosteroids are the most often prescribed initial induction therapy in BS patients. However, corticosteroids alone have poor disease control and an undesirable long-term side effect profile. Combining it with other immunosuppressive agents, such as azathioprine, MTX, hydroxychloroquine, biological drugs such as TNF-α, and IL-1 inhibitors, have been demonstrated to control the course of the disease (29–31). In our case, she was treated with prednisone and MTX as a case of JIA during the last year, but her symptoms were not well-controlled. Then, she was started on TNF-α inhibitor Enbrel subcutaneous injection for 4 months. Since that time, she has shown partial improvement, but her swelling has persisted. Afterward, she was given intra-articular triamcinolone acetonide injections in each wrist and ankle joint, and she continued on low-dose prednisone. Hence, triamcinolone acetonide is an intra-articular steroid preparation, is locally acting, and works only in the joint not in the bloodstream; therefore, it is not related to her renal failure.

As mentioned above, the classical triad of BS may appear in different timeframes, and in some cases, it might not be present at all. In our case, she had never shown symptoms of the skin or ocular involvement and had just developed arthritis. As a result, the patient has been misdiagnosed as having JIA. Therefore, when JIA cannot explain the presentation of the patient and the management effect is not ideal, we should reconsider whether the diagnosis is accurate, particularly if the patient has refractory JIA, as in our case. A thorough family history and further investigations, such as genetic testing are considered essential.

## Data Availability

The original contributions presented in the study are included in the article/Supplementary Material, further inquiries can be directed to the corresponding author.
